# A rare cause of dysphagia due to esophageal intramural pseudodiverticulosis: a case report and review of literature

**DOI:** 10.1186/s12876-020-01209-y

**Published:** 2020-03-16

**Authors:** Osman Ali, Hazel Asumu, Tanisha Kaur, Angelina Mathew, Raymond Kim

**Affiliations:** 1 Department of Internal Medicine, University of Maryland Medical Center Midtown Campus, Baltimore, MD USA; 2 Department of Gastroenterology and Hepatology, University of Maryland School of Medicine, Baltimore, MD 21201 USA

**Keywords:** EIP, Esophageal intramural pseudodiverticulosis, Esophageal stricture, Dysphagia, Dilatation therapy, H2-blocker

## Abstract

**Background:**

Esophageal intramural pseudodiverticulosis is an uncommon, idiopathic disorder characterized by multiple small outpouchings protruding from the esophageal lumen. Esophageal intramural pseudodiverticulosis is associated with conditions such as gastroesophageal reflux disease and diabetes mellitus, as well as emergent complications including pneumomediastinum. The most common presenting symptom is dysphagia with associated esophageal stricture formation. While the pathogenesis of EIP has yet to be determined, it is important to bring awareness to this unique disease with distinctive diagnostic findings and treatment options.

**Case presentation:**

In this case, we present a 62-year-old woman who suffered from dysphagia, an inability to tolerate a regular diet, and unintentional weight loss for several years prior to her diagnoses. She was diagnosed by esophagram and esophagogastroduodenoscopy to have esophageal intramural pseudodiverticulosis, complicated by severe stricture formation. Following treatment with sequential dilatation and maintenance H2-blocker therapy, she achieved significant symptomatic improvement.

**Conclusions:**

This case highlights the importance of accurate identification and treatment of an uncommon cause of dysphagia, esophageal intramural pseudodiverticulosis. Treatment includes dilatational therapy, as successfully demonstrated in our patient. Furthermore, treatment is focused on optimizing medical management, as demonstrated in our patient with the addition of an H2-blocker for GERD, or addressing potentially serious underlying causes, such as carcinoma, with surgery.

## Background

Esophageal intramural pseudodiverticulosis (EIP) is an uncommon disorder distinguished by characteristic pseudodiverticula extending through the esophageal lumen to the outer wall of the esophagus [1–3]. EIP was first illustrated in 1960 by Mendl et al., however, the etiology still remains unclear [4]. Review of 14,350 esophagrams by Levine et al., revealed evidence of EIP in 0.15% [2]. EIP has a bimodal distribution, peaking in both the early teenage years, and in the 6th and 7th decades with a predilection for males [3, 5–7]. The most common symptom of EIP is intermittent or progressive dysphagia with associated esophageal stricture formation, which is appreciated on esophagogastroduodenoscopy (EGD) [3]. Previous literature have reported EIP to be associated with systemic inflammatory conditions, malignancy, and medical emergencies [8, 9]. The current treatment for EIP is focused on addressing the underlying condition and if indicated, endoscopic dilatation therapy.

## Case presentation

**Fig. 1:**
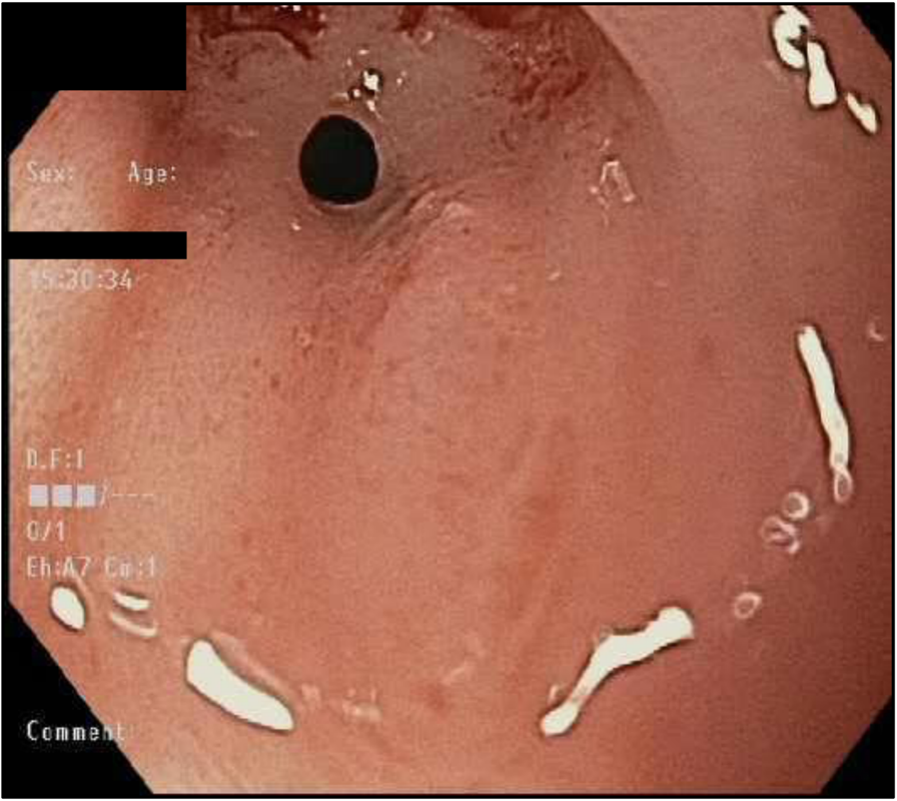
Stricture measuring 3 mm in upper portion of the esophagus

**Fig. 2:**
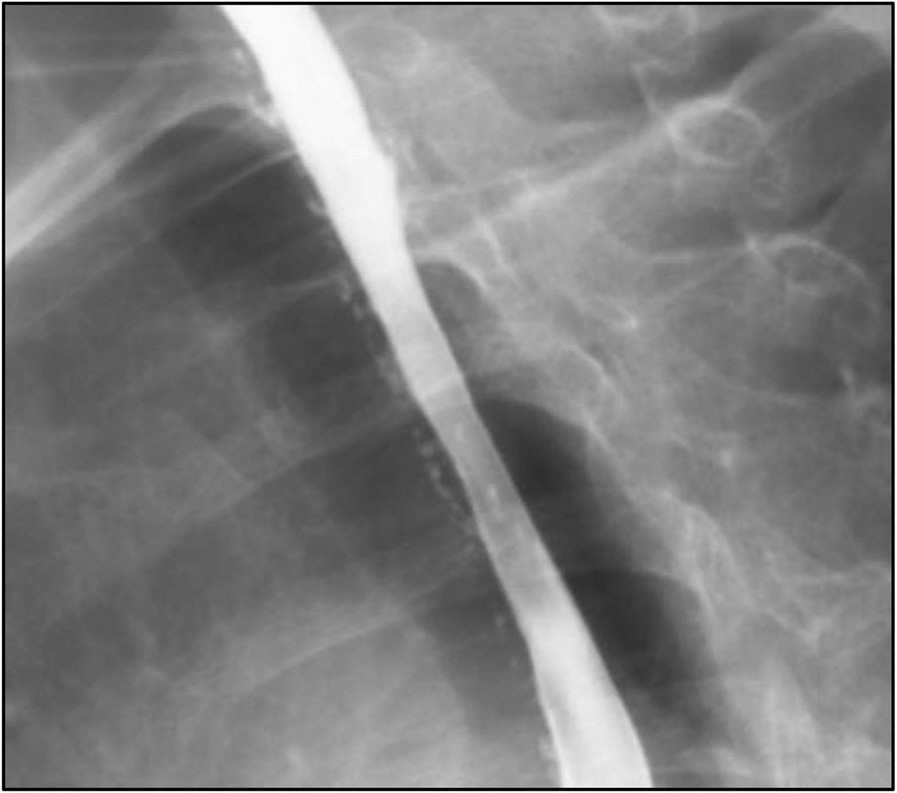
Numerous small submucosal collections of contrast throughout the esophagus

**Fig. 3:**
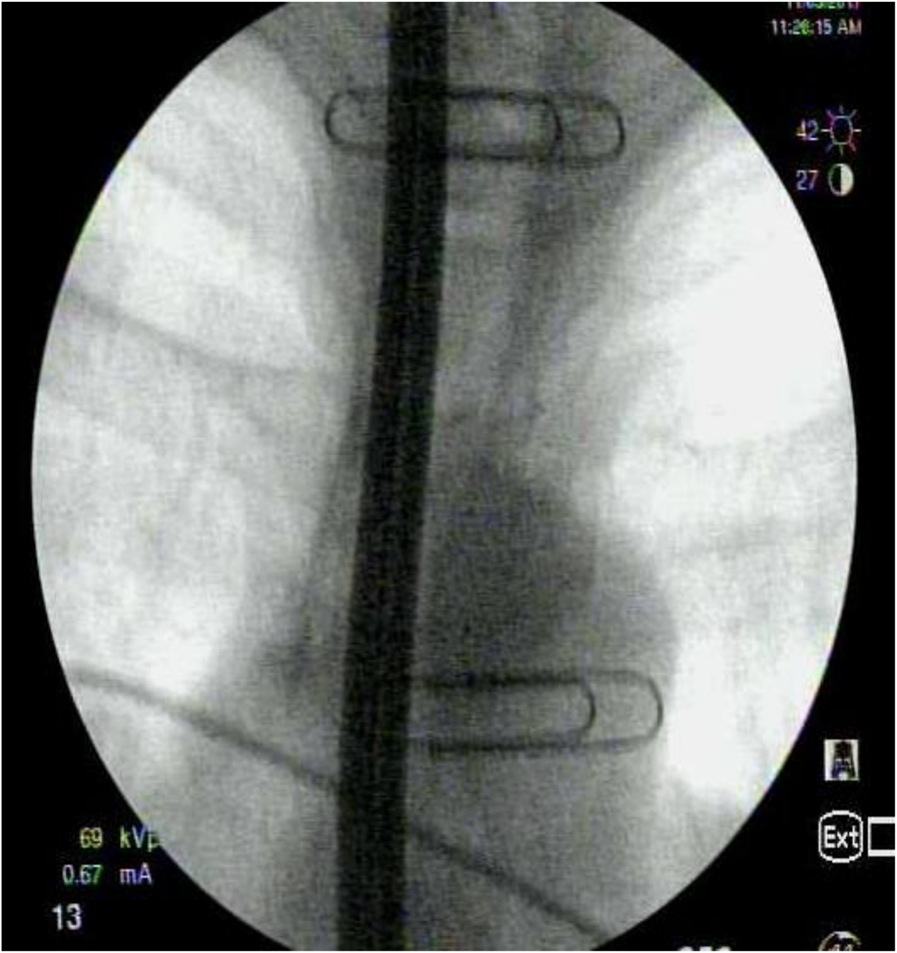
Post dilatation

**Fig. 4:**
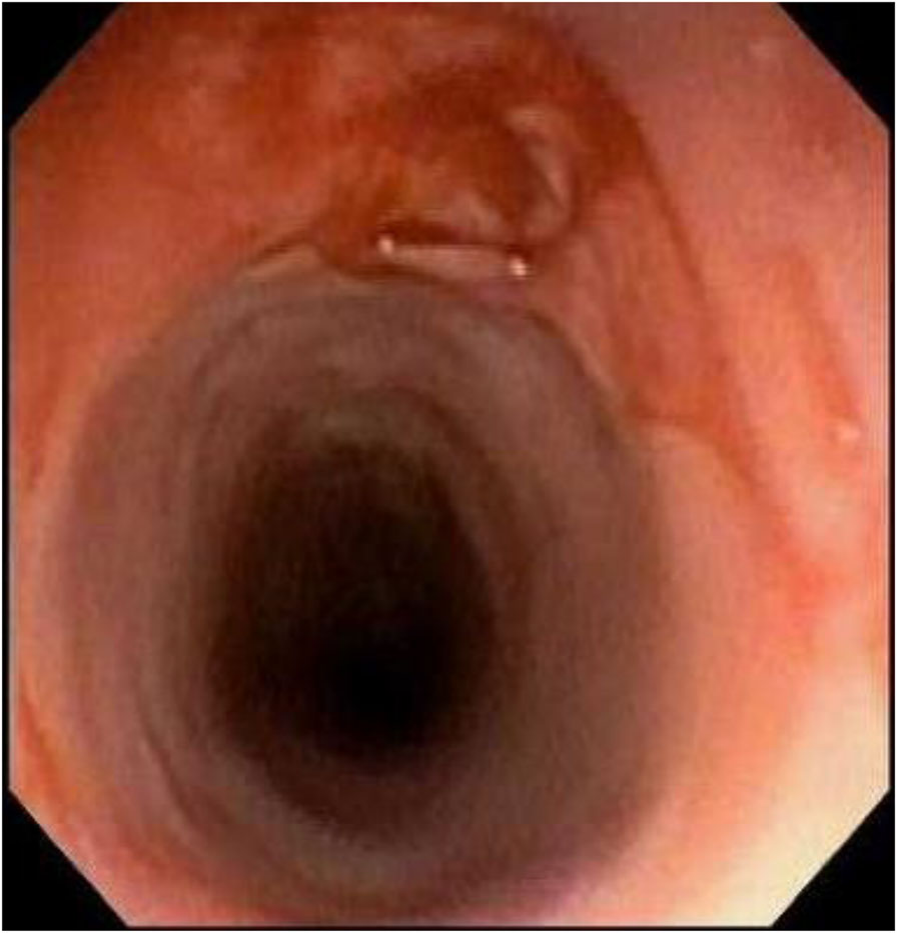
Post dilatation with 27 Fr under fluoroscopic guidance

**Fig. 5:**
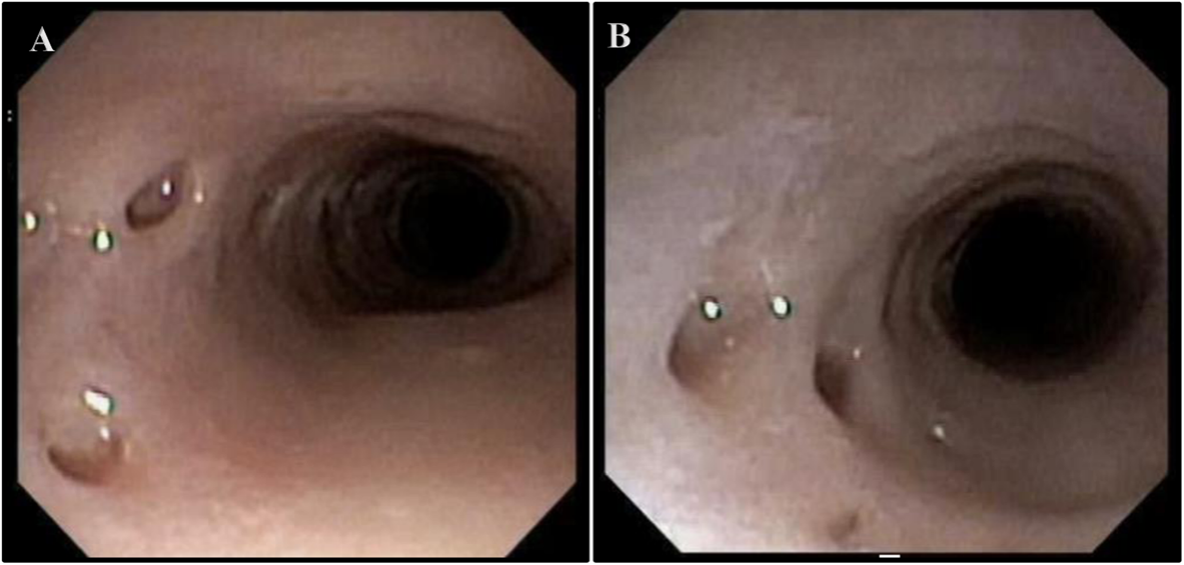
**a** Proximal portion - Pseudodiverticulosis in upper esophagus from 17 cm to 24 cm. **b**. Distal portion - Pseudodiverticulosis in upper esophagus from 17 cm to 24 cm

A 62-year-old female presented with nausea, vomiting, melena, and left lower extremity pain. Her medical history was significant for peripheral vascular disease, liver cirrhosis, chronic pancreatitis, and gastroesophageal reflux disease (GERD). She had a 25 pack-year smoking history, and a prior history of chronic alcohol use. Physical exam revealed a thin, frail, and malnourished woman in overall poor health. Upon initial questioning, she endorsed dysphagia with recurrent gagging, regurgitation of solid food, and unintentional weight loss for over 5 years. She denied any pain with mastication, or odynophagia, but for the last 2 years, she had mostly been restricted to a pureed diet as a result of her symptoms. Additionally, her family history was significant for colon cancer. The initial laboratory exams exhibited an elevated aspartate aminotransferase (71 u/L), alanine aminotransferase (122 u/L), alkaline phosphatase (356 u/L), and a low hemoglobin (5.6 g/dL). EGD and colonoscopy were planned for workup of her anemia, melena, and dysphagia. Initial EGD using GIF HQ 190 (Olympus, Tokyo, Japan) displayed severe stenosis in the upper portion of the esophagus due to a stricture measuring 3 mm in diameter (Fig. 1). The esophageal stricture was subsequently dilated using a 5.5 cm long, 8–10 mm CRE Wireguided Ballon Dilatation Catheter (Boston Scientific, Marlborough, MA) to 8 mm. However, significant narrowing distal to the stenosis was discovered and it was noted that the stricture was longer than 5.5 cm, therefore, the endoscope could not be advanced to measure the stricture length. At this point, the EGD was aborted and barium esophagram was ordered to determine the extent of the stricture. The esophagram displayed stenosis measuring 7 cm in length along with numerous small collections of contrast in the upper portion of the esophageal submucosa, consistent with EIP findings (Fig. 2). Additionally, colonoscopy performed during the initial workup was negative for a source of bleeding, therefore, her profound anemia was likely secondary to a combination of her poor oral intake, cirrhosis and an open, weeping ulcer on the foot. A repeat EGD was performed for subsequent dilatation with a Savary-Gilliard dilator (Cook Medical, Bloomfield, IN), 24 French (Fr) and 27 Fr dilation was completed without complications (Figs. 3 and 4). Post dilation stenosis was measured in the upper third of the esophagus from 17 cm to 24 cm from incisors (Fig. 5a, b). A total of two sessions of dilatation therapy were performed during her hospitalization and were tolerated well. She was sent home on a proton-pump inhibitor (PPI) and within 4 weeks switched to a Histamine-2 (H2) receptor antagonist due to persistent hypomagnesemia. Three weeks after dilatation, on follow-up examination, she reported significant improvement in her dysphagia and was tolerating a full regular diet for the first time in 2 years. A repeat endoscopy was not indicated at follow-up examination as she reported no dysphagia or related issues. The patient was contacted 2 years later and reported no recurrence of dysphagia while tolerating a full solid and liquid diet.

## Discussion and conclusions

Although idiopathic, two mechanisms of possible injury contributing to the structural and pathological findings seen in EIP predominate. Chronic inflammation resulting in obstruction of the excretory ducts and chronic irritation of the esophagus causing fibrosis of the submucosa [10]. The risk of developing EIP are increased in patients who have coexisting diseases, such as, HIV, diabetes mellitus, esophageal candidiasis, chronic alcohol abuse, Mallory-Weiss syndrome, Crohn’s disease, GERD, and corrosive esophageal injury [4, 11–14]. Abnormalities in esophageal motility, including, uncoordinated peristalsis, Jackhammer esophagus, hypoperistalsis, and achalasia, have also been associated with EIP [15, 16]. In the case of our patient, malignancy was a priority differential diagnosis given her family history of unspecified colonic carcinoma, weight loss, anemia and high-risk comorbidities. Importantly, previous reports have demonstrated a statistically significant difference between the prevalence of EIP in patients with esophageal cancer and of those without esophageal cancer [17]. Plavsic et al., retrospectively reviewed 245 patients with esophageal carcinoma compared to a control group of 6400 esophagograms obtained for indications other than esophageal carcinoma. Intramural pseudodiverticulosis of the esophagus was found in 11 patients with esophageal carcinoma (4.5%) and in 6 control subjects (0.09%). The prevalence of EIP was significantly higher in patients with esophageal carcinoma when compared to patients who underwent esophagograms for other indications (*p* < 0.0002), suggesting an association for increased risk of esophageal carcinoma in patients with EIP. Although no guidelines have been published, periodic surveillance of patients with intramural pseudodiverticulosis of the esophagus for esophageal carcinoma is advised [17]. Additionally, EIP has been associated with life threatening conditions, as suggested in two case reports of critically ill patients. EIP was the proposed underlying cause of increased intraluminal pressure leading to esophageal perforation and pneumomediastinum in those two cases [5, 18]. Of note, a study consisting of 368 patients post esophageal dilatational revealed no perforation in the 4 patients with pseudodiverticulosis [19]. However, perforation is a reported complication of EIP and treatment with dilatational therapy should be carefully considered as increases in intraluminal pressure, such as in vomiting, may increase risk of perforation [20].

Radiologic imaging is the primary modality for the diagnosis of EIP, a single contrast barium swallow examination is the study of choice, as the thin barium enters pouches better than the higher density agent used in double contrast studies [2, 21]. EIP is characterized by numerous, 1–4 mm, flask shaped diverticula, which represent outpouchings from the esophagus and may appear to float adjacent to the esophageal wall [2, 21]. These outpouchings are segmented in the majority of cases but may also be diffuse throughout the esophagus [21]. Intramural tracking can also be visualized on barium swallow as linear tracks, suggestive of pseudodiverticula interconnecting and bridging to one another. Cases of fistulisation, abscesses, as well as tract and sinus formations have been reported in infectious causes of EIP [22–24]. However, scant literature exists regarding the prevalence of infectious causes of EIP. It is further postulated that in some cases, underlying polymicrobial infection may be the cause of EIP and reports have shown symptomatic improvement of patients after empiric antimicrobial therapy [25, 26]. It is important to be aware of these possible findings in order to characterize and treat the pseudodiverticula especially when caring for immunocompromised patients.

Thus far, serial endoscopic dilatation seems to be the mainstay of treatment for symptom relief. It has been shown to relieve the symptoms of dysphagia that are associated with esophageal stenosis and strictures. An observational study of 21 patients with EIP requiring initial therapy totalling 103 dilatations sessions (pooled mean 6.1 initial dilations per patient) revealed symptom recurrence in 12 of the 21 cases which required additional repeat dilations on follow-up despite initial therapy. Of note, the majority of these patients required an average of 9 dilatational sessions per patient (range 2–25) during follow-up [27]. Therefore, although dilatation therapy relieves symptoms temporarily, it may not be the ultimate treatment for EIP, as the pseudodiverticula are still present in many cases. Surgical intervention can be considered in patients with severe strictures and when medical management or dilatational therapy is not sufficient in providing symptom relief. One case report showed that an esophagectomy improved severe dysphagia [28]. Another report presented a patient who had severe strictures, which eventually led to aspiration pneumonia. This patient successfully underwent a thoraco-laparoscopic esophagectomy, after conservative management with antifungals did not improve his symptoms [29]. Therefore, esophagectomy is beneficial in preventing further complications that could arise as a result of EIP. In addition to dilatation therapy and esophagectomy, treatment should be focused on managing comorbid conditions and underlying causes. Previous reports have focused on the use of PPI as part of the treatment for EIP, especially in patients with underlying GERD. In this case, our patient has a history of GERD and a contraindication to proton pump inhibitors, therefore, an H2-receptor antagonist was added to the regimen with successful results. The addition of sucralfate, alongside dilational therapy, is another option in patients that are unable to tolerate proton pump inhibitors as it has been shown to relieve symptoms of dysphagia [30]. Smoking cessation as well as discontinuation of other offending agents should be encouraged. Periodic endoscopy is recommended due to the association of EIP and esophageal cancer [17]. In conclusion, we report a case of a patient with EIP whose dysphagia of several years was successfully treated after only 2 simultaneous sessions of dilation therapy during hospitalization and has remained symptom free while on maintenance H2-receptor blocker therapy. This case expands upon the potential associations of EIP, current therapy of EIP, and emphasizes the importance of investigating the serious underlying causes and complications associated with EIP.

## Data Availability

Not applicable.

## Abbreviations

EGD: Esophagogastroduodenoscopy
EIP: Esophageal intramural pseudodiverticulosis
Fr: French
GERD: Gastroesophageal reflux disease
H2: Histamine-2
PPI: Proton pump inhibitor
TTS: Through the scope
